# How Dispersion Interactions at the Excited State Can Tune Photochromism of Embedded Chromophores

**DOI:** 10.1021/jacs.5c19241

**Published:** 2025-12-24

**Authors:** Ciro A. Guido, Lorenzo Cupellini, Benedetta Mennucci, Carles Curutchet

**Affiliations:** † Dipartimento di Scienze e Innovazione Tecnologica, 19050 Universitá del Piemonte Orientale, Viale T. Michel 11, Alessandria 15121, Italy; ‡ Dipartimento di Chimica e Chimica Industriale, 9310 Universitá di Pisa, Via G. Moruzzi 13, Pisa 56124, Italy; § Departament de Farmàcia i Tecnologia Farmacèutica, i Fisicoquímica, Facultat de Farmàcia i Ciències de l’Alimentació, Universitat de Barcelona (UB), Barcelona 08028, Spain; ∥ Institut de Química Teòrica i Computacional (IQTCUB), 16724 Universitat de Barcelona (UB), Barcelona 08028, Spain

## Abstract

We present QM/MMPol-cLR^3^, a polarizable embedding quantum mechanics/molecular mechanics (QM/MM) framework that includes explicit, state-specific dispersion terms. This method enables a rigorous treatment of dispersion on top of electrostatic and induction effects in ground- and excited-state calculations. Using QM/MMPol-cLR^3^, we show that dispersion interactions control excited-state solvatochromism through two distinct mechanisms. In azulene, opposite shifts of the L_a_ and L_b_ states arise from state-specific dispersion linked to changes in excited-state polarizability. In bacteriochlorophyll a, dispersion instead stems from the interplay between polarizability changes and transition-dipole-driven response, governing the *Q*_*y*_ and *Q*_*x*_ shifts. Finally, application to the LH2 complex reveals pigment-dependent dispersion shifts between the B800 and B850 rings, impacting the excitation-energy transfer. These results establish dispersion as an essential, nonempirical component for predictive excited-state simulations in complex environments.

## Graphical abstract

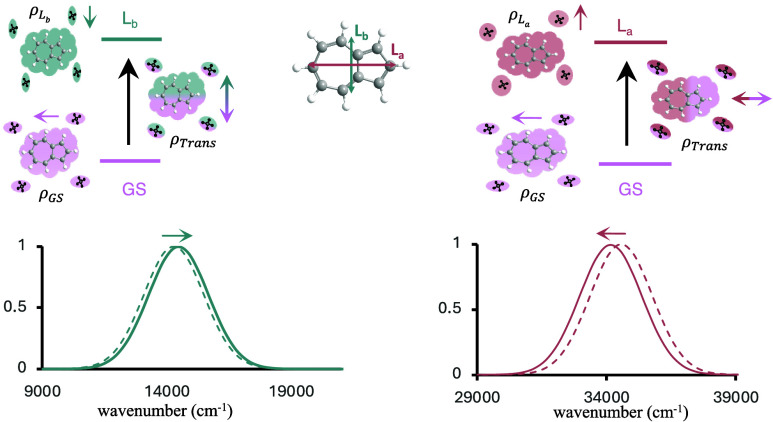

## Introduction

Understanding how intermolecular interactions shape molecular processes is central to modern chemistry, materials science, and biophysics. Among them, van der Waals (vdW) dispersion forces arising from correlated quantum fluctuations of the electron density play a decisive role across length scales, from cohesion in molecular crystals to recognition in biomolecular assemblies. Although often labeled as “weak,” dispersion contributes substantially to binding, structure, and reactivity, and has been shown to determine mechanical and spectroscopic signatures of both materials and biological systems.^1–4^ At the electronically excited state, dispersion acquires a distinct relevance, especially in nonpolar environments,^5–8^ revealing unusual solvatochromic responses and photophysical behaviors that cannot be explained by electrostatics alone.^9–14^ The theoretical methods addressing the role of vdW interactions involving electronic excited states remain scarce. Although sophisticated schemes which capture London dispersion effects proper to excited states (including repulsive dispersion) were developed already during the 90’s,^15–17^ their application has been limited to atoms or small molecular systems. In fact, these methods require a fairly complex quantum electrodynamical treatment of the vdW-Casimir potential. These approaches have found a renewed interest, and very recently, Pernal and co-workers showed that an additional term appears in the Casimir–Polder expression for dispersion interactions if one molecule is in the excited state.^18^ They developed a generalized Casimir–Polder expression valid for excited states^18^ and implemented it within a multireference treatment.^19^

Only few studies have considered computational procedures to include dispersion effects for excited states: for example, the local response dispersion (LRD) model of Ikabata and Nakai,^20,21^ which was used to calculate interaction energies of exciton-localized molecular complexes from the S66 ^22^ benchmark set as well as molecular excimers; the empirical variation of the density functional theory (DFT)-D2 dispersion coefficients for the small model ethene–argon and formaldehyde–methane complexes of Briggs and Besley;^23^ or the work of Johnson and collaborators on dispersion coefficients within the exchange-hole dipole moment (XDM)^24^ model for excited states of some conjugated hydrocarbons, pull–pull chromophores, and CT complexes.^25^ More recently, Goerigk and co-workers showed that TD-DFT methods (even dispersion-corrected) struggle with the description of noncovalent interactions in excited states.^12,26^ Barcza et al. proposed and benchmarked dispersion interaction corrections for excited states in fragment-based methods.^27^

Examples become even more scarce in complex systems characterized by increasing degrees of freedom. Their size in fact prevents the use of full-quantum mechanical (QM) methods, and multiscale QM/classical approaches instead become necessary.

Some strategies have been proposed in the context of continuum solvation models. Empirical expressions have been derived in the dipolar approximation by Renger et al.^28^ and Marenich et al.^29^ The latter method is called “Solvation Model with State-Specific Polarizability” (SMSSP). Within the polarizable continuum model (PCM^30^), the dispersion model by Amovilli and Mennucci^31^ has been extended to electronic excitations.^32,33^ A different method proposed by Amovilli and Floris is based on the measure of the electronic electric field fluctuations by means of quantum Monte Carlo approaches.^34^ Finally, one of the present authors has introduced a reformulation of the problem of a quantum solute in a polarizable environment in terms of open quantum systems (OQS) theory,^35^ showing that both polarization and dispersion interactions between the molecular subsystem and the environment naturally arise in a time-dependent stochastic Schroedinger equation (TD-SSE) treatment and should be included together to properly describe processes at both the ground and excited states. In line with this, a simple but effective model has been proposed,^36^ called cLR^2^, which approximates the state-specific polarization effects and part of solute–solvent dispersion^35^ within a TD-DFT/PCM framework.

On the other hand, in the context of atomistic QM/MM frameworks, nonelectrostatic interactions between the QM and MM subsystems are typically retained only at the MM level and described through empirical analytical functions such as Lennard-Jones potentials. Recently, polarizable embedding QM/MM approaches that explicitly account for both dispersion and exchange–repulsion have been proposed for ground-state systems.^27,37,38^ Here, we extend these developments to the excited-state regime by introducing an effective, state-specific polarization and dispersion QM/MM model applicable to systems of increasing environmental complexity, from isotropic solvents to biological matrices. The approach (from now QM/MMPol-cLR^3^) builds upon the cLR^2^ scheme, which captures state-specific polarization and part of the solute–solvent dispersion, and combines it with the dispersion model previously developed by some of the present authors for ground-state interactions.^37^ This latter component follows the strategy originally proposed within density functional theory by Tkatchenko and Scheffler (TS).^39^

We demonstrate that the QM/MMPol-cLR^3^ model successfully reproduces and rationalizes solvatochromic shifts that cannot be captured by electrostatics or mutual polarization alone. As representative cases, it accounts for the counterintuitive spectral trends observed in azulene^7^ and in bacteriochlorophyll a dissolved in low-polarity solvents.^28^ The analysis reveals that shifts of the lowest electronic excitations originate from distinct dispersion mechanisms between the chromophore and its environment, governed, respectively, by excited-state polarizability and transition density. In addition to reproducing experimental observations, the method is benchmarked against full-quantum reference calculations, confirming its accuracy and physical consistency. Finally, we show that dispersion interactions can directly modulate excitation-energy transfer in multichromophoric assemblies, as evidenced by the differential shifts of pigment excitation energies in the B800 and B850 bands of the light-harvesting 2 (LH2) complex.^40–42^

## Theory

The model introduced is rooted in the open quantum system description of a molecular quantum subsystem embedded in a polarizable environment.^35^ Within a non-Markovian stochastic Schrödinger equation treatment,^43^ the coupled and time-retarded electron dynamics of the system and its environment can be shown to decompose naturally into three contributions: (i) a state-specific response of the environment polarization, (ii) a state-specific dispersion term arising from the environment’s response to fluctuations of the molecular electronic density, and (iii) a symmetric counterpart corresponding to the molecular response to fluctuations in the environment’s electronic degrees of freedom. A conceptual picture of the three terms is reported in Figure 1. The last term can be explicitly included by a stochastic treatment in a time-dependent picture. Alternatively, when a stationary-state framework is adopted, this missing contribution should be approximated by an effective treatment that ensures its influence is properly retained. In both continuum and atomistic polarizable models, the transition energies computed using a linear response (LR) scheme for the environment response contain a term that can be categorized as dispersion rather than a polarization effect.^44–47^ However, only very recently, some of us proved that such an LR term is indeed an approximation to the solute response-environment fluctuation contribution.^35^ Within a real-frequency treatment^48^ of the intermolecular interaction theory of McWeeny,^49^ additional terms appear.^35^

**Figure 1:**
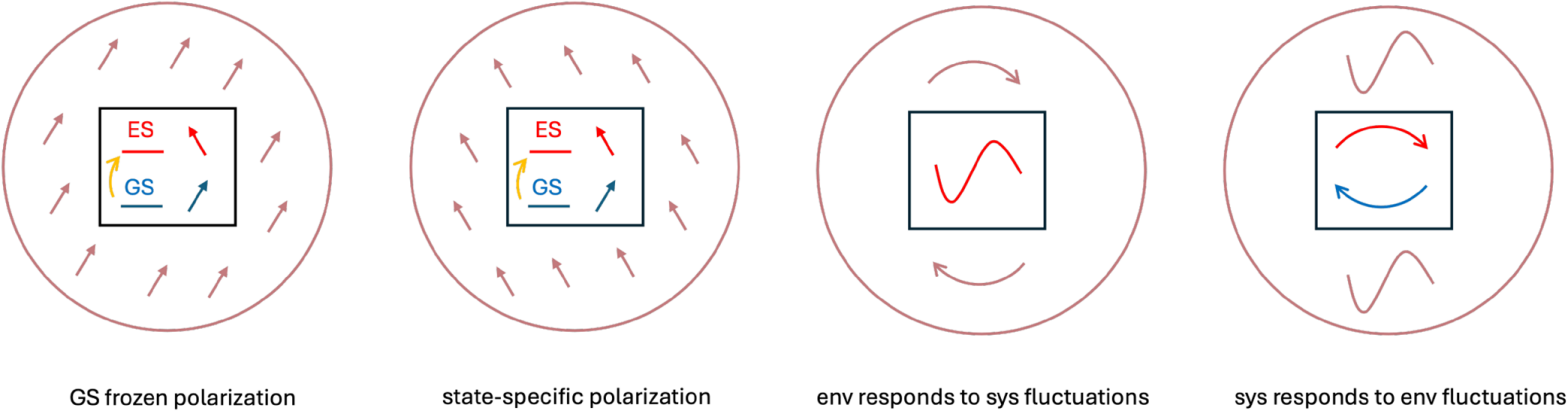
Pictorial representation of the components of solute-environment interactions, as obtained in an OQS treatment of a bath of polarizable charges^35^

Our QM/MMPol-cLR^3^ scheme is therefore based on the use of: (i) a state-specific correction due to the environment polarization (*R*_SS–pol_) by the cLR approach, (ii) a QM/MMPol linear response term (*R*_LR–disp_) that approximates the solute-environment fluctuation dispersion, and (iii) a state-specific dispersion term (*R*_SS–disp_). The QM/MMPol-cLR^3^ transition energy, therefore, adds up these three contributions to the excitation energy computed in the presence of an environment with a polarization frozen to the ground state (Δ*E*_GS–pol_):

1 $$\Delta E_{\mathrm{cL}R^{3}}=\Delta E_{\mathrm{GS}‐\mathrm{pol}}+R_{\mathrm{SS}‐\mathrm{pol}}+R_{\mathrm{LR}‐\mathrm{disp}}+R_{\mathrm{SS}‐\mathrm{disp}}$$

The last term is the difference between the excited and ground-state dispersion contributions:

2 $$R_{\mathrm{SS}‐\mathrm{disp}}=R_{\mathrm{ES}‐\mathrm{disp}}-R_{\mathrm{GS}‐\mathrm{disp}}$$

The LR (i.e., Δ*E*_GS–pol_ + *R*_LR–disp_) and the cLR (i.e, Δ*E*_GS–pol_ + *R*_SS–pol_) schemes have been largely discussed both in literature for both continuum^36,50,51^ and atomistic^52,53^ polarizable models. Here, we just recall that the first is a functional of the transition density, whereas the second is a functional of the ground-to-excited state density difference. Let us focus here on the new state-specific dispersion term, *R*_SS–disp_. This is based on our GS protocol to determine the dispersion term *R*_GS–disp_, whose details are reported in ref 37. Here, we just recall that the dependence of the dispersion energy on the atomic polarizabilities is described by taking advantage of the approximate relation between polarizability and volume.^39^ As a result, the pairwise additive dipolar interaction between two atoms of a given system, which goes with the inverse sixth power of the distance, becomes density-dependent:

3 $$R_{\mathrm{ES}‐\mathrm{disp}}=-\sum_{A}^{\mathrm{QM}}\sum_{B}^{\mathrm{MM}}f_{\mathrm{AB}}\left[\gamma_{A}^{\mathrm{ExS}}\right]C_{6,\mathrm{AB}}\left[\gamma_{A}^{\mathrm{ExS}}\right]R_{\mathrm{AB}}^{-6}$$

Here, the sum runs over all pairs of atoms of the QM (A) and MM (B) subsystems, where *R*_AB_ is the interatomic distance between atoms A and B, *C*_6,AB_ is the effective dipolar coefficient, and *f*_AB_ is a damping function to correct for the divergence at short distances.

The effective *C*_6_ coefficients for an atom inside a molecule can be written as^39^

4 $$C_{6,\mathrm{AB}}^{\mathrm{eff}}=\frac{2C_{6,\mathrm{AA}}^{\mathrm{eff}}C_{6,\mathrm{BB}}^{\mathrm{eff}}}{\frac{\alpha_{B}}{\alpha_{A}}C_{6,\mathrm{AA}}^{\mathrm{eff}}+\frac{\alpha_{A}}{\alpha_{B}}C_{6,\mathrm{BB}}^{\mathrm{eff}}}$$

Assuming that atomic polarizabilities α_A_ are proportional to the atomic volume,

5 $$\frac{C_{6,\mathrm{AA}}^{\mathrm{eff}}}{C_{6,\mathrm{AA}}^{\mathrm{free}}}\approx {\left(\frac{\alpha_{A}}{\alpha_{A}^{\mathrm{free}}}\right)}^{2}\approx {\left(\frac{V_{A}}{V_{A}^{\mathrm{free}}}\right)}^{2}=\gamma_{A}^{2}$$

Therefore, the first two terms in eq 3 depend on the QM electron density through the γ_A_ atomic volume ratios, obtained by dividing the effective atomic volume in the molecule *V*_A_ and that of the free atom *V*_A_^free^ *:*

6 $$\gamma_{A}\left[\rho \left(r\right)\right]=\frac{V_{A}\left[\rho \left(r\right)\right]}{V_{A}^{\mathrm{free}}}=\frac{\int r^{3}w_{A}\left(r\right)\rho \left(r\right)dr^{3}}{\int r^{3}\rho_{A}^{\mathrm{free}}\left(r\right)dr^{3}}$$

The last equation uses the Hirshfeld weight (*w*_A_) to partition the molecular electron density into atomic contributions.^54^ Instead, for MM atoms, γ_B_ values can be precomputed from TS QM calculations or directly estimated from the MMPol polarizabilities.^37^

The extension of this approach to treat electronically excited chromophores obviously implies the use of the excited-state density^17,18,35,44^ in (eqn [Disp-formula eq6]) to be used for (eqn [Disp-formula eq3]). However, the assumption within the TS approach that atomic polarizabilities are proportional to volume^39^ is only valid for a ground state. The free atom is in fact an inadequate reference for describing an excited state delocalized over a molecule. As clarified in the seminal analyses of Power and Thirunamachandran,^15–17^ and later emphasized by Pernal and co-workers,^18^ this inadequacy originates from the additional term that appears in the expression of polarizability when the molecule is electronically excited. This term involves negative-frequency transitions that disrupt the usual connection between polarizability and volume. Consequently, repulsive dispersion interactions may arise because the imaginary frequency-dependent polarizability of an excited molecule can even become negative, an effect that has no analogue in terms of an atomic volume, which cannot take negative values.

To correct the definition of γ_A_^ExS^, we introduce an atom-dependent rescaling factor to take into account the site-specific changes in polarizability following the electronic transition. To this end, we adopt the method developed by Heid, Hunt, and Schröder (HHS) for decomposing ground and excited-state molecular polarizabilities into atomic contributions^55^ based on the use of the full QM electronic density and the Stone’s distributed multipole analysis.^1,56^ This procedure has the advantage of reproducing the molecular polarizability as the sum of the atomic contributions (see Table 1 in the Results and Discussion section). Since we want to incorporate to our ground-state parametrization the variation of the molecular polarizability following the excitation, we impose that the scaled excited-state atomic polarizability (α̃_A_^ExS^ = α_A_^ExS^·β_A_) is able to reproduce the ratio between the ground and the excited states obtained by the HHS method, i.e.,

7 $$\frac{{\mathrm{\alpha ̃}}_{A}^{\mathrm{ExS}}}{\alpha_{A}^{\mathrm{GS}}}=\frac{\alpha_{A}^{\mathrm{HHS},\mathrm{ExS}}}{\alpha_{A}^{\mathrm{HHS},\mathrm{GS}}}\Rightarrow \beta_{A}=\frac{\alpha_{A}^{\mathrm{GS}}}{\alpha_{A}^{\mathrm{ExS}}}\cdot \frac{\alpha_{A}^{\mathrm{HHS},\mathrm{ExS}}}{\alpha_{A}^{\mathrm{HHS},\mathrm{GS}}}$$

These scaling factors are therefore applied, for an excited state, to the volume ratio parameters γ_A_ used in (eqn [Disp-formula eq3]):

8 $${\mathrm{\gamma ̃}}_{A}^{\mathrm{ExS}}=\gamma_{A}\left[\rho^{\mathrm{ExS}}\left(r\right)\right]\cdot \beta_{A}$$

We note that the model retains the explicit density-dependence of ExS dispersion by computing the atomic volume ratios, γ_*A*_[ρ^ExS^(*r*) ], using the excited-state density. Thus, one needs to account for the changes in TS polarizabilities using α_A_^GS^ and α_A_^ExS^ in eq 7. We chose to keep the full density-dependence for consistency between GS and ExS dispersion terms. Alternatively, one could neglect the changes in atomic volume ratios in the excited state and obtain β_A_ directly from the ratio of HHS ExS and GS polarizabilities.

**Table 1:** Molecular Polarizabilities (α_mol_, au) of Different Electronic States of Azulene Obtained as the Sum of Atomic Contributions^a^

	α_mol_^GS^	α_mol_^L_b_^	α_mol_^L_a_^
QM/MMPol-TS	125.0	125.6	126.4
QM/MMPol-cLR^3^	125.0	120.0	135.1
full DFT	125.4	119.6	135.6

a The QM/MMPol-cLR^3^ values are obtained from the QM/MMPol-TS ones by the scaling procedure described in the text using gas-phase HHS polarizabilities. Full DFT results refer to analytical TD-DFT/M062X simulations in the gas phase.

The computational procedure includes a preliminary step to parametrize a given excited state of the molecule of interest, which involves computing the HHS polarizabilities of ground and excited states of the QM subsystem, as described in ref 55, to obtain the scaling factors. The β_A_ factors can be obtained by HHS polarizabilities computed in the gas phase or for the specific environments; this last procedure requires, of course, a statistical averaging over different configurations. However, only small changes are observed for rigid solutes in isotropic media; see the Supporting Information.

Finally, we note that this treatment assumes that the electronic excitations of the environment occur at significantly higher energies with respect to the molecular subsystem of interest. Otherwise, the excitation would be delocalized on environment molecules as well, and a molecule–environment separation would not be possible. However, such cases can be treated within an exciton model, which treats all nearly resonant excitations on the same basis.

We implemented the QM/MMPol-cLR^3^ method in a locally modified development version of the Gaussian suite of programs.^57^ The main source of additional computational effort relative to a standard QM/MMPol-cLR calculation lies in the self-consistent implementation of the TS-vdW dispersion–repulsion operator for the ground state. In this formulation, all density-dependent TS-vdW quantities (Hirshfeld weights, atomic volume ratios γ_A_, and their functional derivatives) enter the effective Kohn–Sham (KS) operator and must be iteratively updated until convergence, as detailed in ref 37. This ground-state SCF cycle can be a computationally demanding step, but a post-KS correction is also possible:^37^ in this case, the TS-vdW dispersion–repulsion energy is computed at the end of the SCF cycle as a correction. We point out that once the scaling factors and the GS quantities are determined, the computational cost of a QM/MMPol-cLR^3^ calculation is similar to a QM/MMPol-cLR simulation, since the state-specific dispersion term *R*_SS–disp_ requires computing the excited-state density (computed by the *z*-vector approach), which is also used to calculate *R*_SS–pol_ of cLR. The *R*_LR–disp_ term is naturally included in the procedure, as an output of solving the TD-DFT QM/MMPol equations.

### Computational Details

We investigated different systems with increasing complexity, as reported in Figure 2. QM/MMPol calculations for the pyridine (QM) – MeNH_2_ (MM), and uracil (QM) – cyclopentane (MM) dimers were performed at the LC-BLYP/6–311++G­(2d,2p) level of theory using geometries taken from the S22 benchmark set. The model of azulene in CCl_4_ was obtained from an MD simulation. Azulene was optimized at the B3LYP/6–31G­(d)//PCM level and inserted into a box of 1520 CCl_4_ molecules. Parameters for azulene and CCl_4_ were derived using the general Amber force field (GAFF).^58^ After minimization and NPT heating, a 10 ns NVT production simulation was performed, and 200 snapshots were extracted, where the azulene geometry was kept frozen. For Bacteriochlorophyll a (BChl-a), we simulated the *Q*_*y*_ and *Q*_*x*_ first singlet states in two different environments: a low-polarity solvent (CCl_4_) and the LH2 antenna complex of purple bacteria. In the first case, BChl-a was solvated in a box with 611 CCl_4_ molecules. After minimization, the system was thermalized up to 100 K in the NVT ensemble and 300 K in the NPT ensemble for 500 ps, and a 1000 ns NVT production run was obtained. MM parameters for BChl-a were taken from the literature.^59^ A total of 160 uncorrelated frames were extracted from the last 800 ns. The solute was left free to move to ensure the sampling of the conformational degrees of freedom. Structures of BChls-a in the LH2 complex from *Rhodoblastus acidophilus* were taken from a previous MD simulation.^60^ Here, we select 50 frames for each of the three nonequivalent BChls-a.

**Figure 2:**
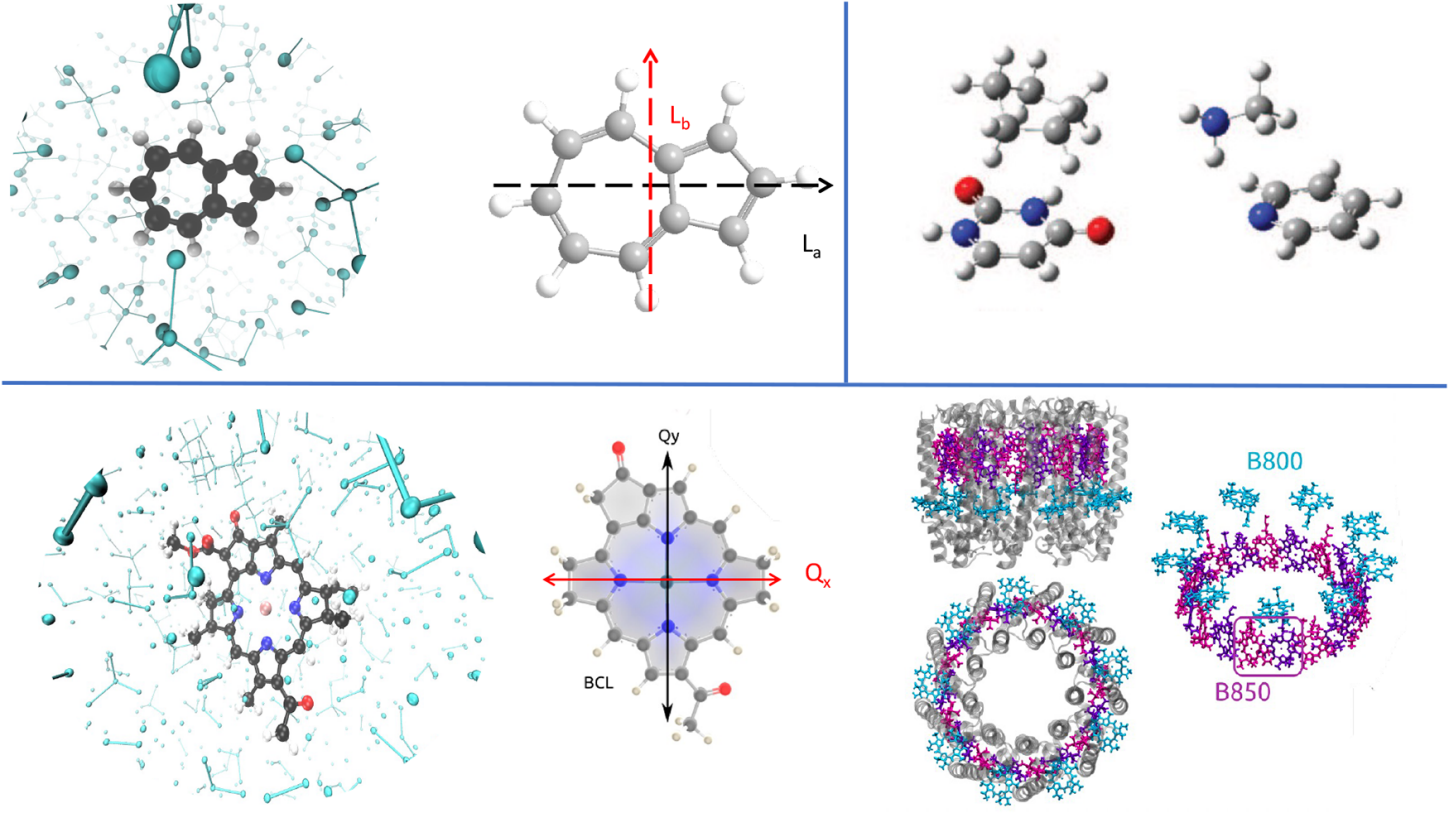
Sketch of the molecular systems considered in this study: azulene in CCl_4_ solvent (top left); uracil-cyclopentane and pyridine-methylamine dimers (top right); bacteriochlorophyll a in CCl_4_ solvent (left) and in the LH2 complex of *R. acidophilus* (right, PDB code: 1NKZ). Note that the three nonequivalent BChls-a are depicted in different colors.

QM/MMPol calculations on azulene and BChl-a were performed at the M06–2*X*/6–31+G­(d) and B3LYP/6–31+G­(d) level of theory, respectively, adopting the Amber pol12 AL force field for the environment.^61^ PBE/6–311++G­(d,p) calculations were used to derive the γ_*B*_ values and polarization-consistent charges for pyridine, MeNH_2_, uracil, cyclopentane, and CCl_4_. For charge fittings, we used the Polchat tool.^62^ For gas-phase dimers, the γ_B_ calculations included the other monomer by using an MMPol description. In the case of LH2, γ_B_ parameters were estimated from the MMPol polarizabilities.^37^ All QM/MMPol calculations were performed by using a polarization cutoff radius of 15 Å. All MD simulations were performed using Amber 2018 software.^63^ Finally, all scaling factors β_A_ of excited-state γs have been obtained from the atomic polarizabilities computed using the procedure and the Python script reported in ref 64 and the GDMA program,^65^ at the same QM level of theory of the corresponding QM/MMPol-cLR^3^ simulation. Gas-phase HHS polarizabilities were computed for pyridine­(QM)-MeNH_2_(MM) and uracil­(QM)-cyclopentane­(MM) since these systems are dimers in vacuo. The same protocol was applied to azulene in CCl_4_, given that the molecular geometry of azulene was held fixed during the MD simulation, and the solvent is isotropic. Calculations based on QM/MMPol in CCl4 for the determination of HHS polarizabilities yield only minor deviations, as detailed in the Supporting Information. In contrast, when the environment is anisotropic, as in the LH2 complex or when the chromophore exhibits substantial structural flexibility, it becomes more reliable to derive the β_A_ scaling factors directly for the specific environment. This second approach was therefore adopted for the BChl-a cases. Although multiple calculations are required to obtain HHS polarizabilities for each distinct environmental configuration, resulting in a more labor-intensive calibration of the β_A_ values, this approach provides a more accurate and internally consistent description of the state-specific dispersion contribution.

We also assessed the sensitivity of the QM/MMPol-cLR^3^ approach to the exchange–correlation (xc) functional and basis set, using azulene in CCl_4_ as an example. The variation is again modest when moving from M062X to CAM-B3LYP (20 cm^–1^ for L_b_ and 50 cm^–1^ for L_a_), consistent with the similar fraction of exact exchange in the two functionals (54% for M062X and from 19 to 65% for CAM-B3LYP). Reducing the exact exchange content with PBE0 leads instead to a more marked decrease of the solvatochromic shift, on the order of 100 cm^–1^ for both excited states, in line with previous observations of fine interplay between state-specific environment response approaches and the xc-functionals used.^46^ Moreover, changing the basis set from a split-valence double-ζ (6–31+G*) to a triple-ζ (6–311+G*) induces only minor effects: 40 cm^–1^ for L_b_ and as little as 8 cm^–1^ for L_a_.

All the required files (molecular structures, the QM and MM γ_A_ parameters, and the applied scaling factors β_A_) to compute the different components of transition energies within the QM/MMPol-cLR3 framework are available in a Zenodo repository.^66^

## Results and Discussion

### Comparison with a full QM-Based Model

We first validate the QM/MMPol-cLR^3^ model by comparison with a full QM model, the LRD approach of Ikabata and Nakai.^20^ We consider two dimers in which one monomer is excited in the presence of a ground-state partner (Figure 2).

The LRD model computes a pairwise dispersion correction using a multipole expansion of the Coulomb operator and the local response approximation to the density response function calculated from the TD-DFT excited-state density. Since LRD is available only for the LC-BOP functional in Gamess US (2023 R2),^67^ which is not implemented in Gaussian,^57^ we employed here the LC-BLYP functional in our calculation, setting the same range-separated parameter (μ = 0.33). We selected an excited pyridine in the presence of a MeNH_2_ molecule at the GS, and an excited uracil in the presence of a GS cyclopentane, employing the same basis set as Ikabata and Nakai, i.e., 6–311++G­(2d,2p).^20^

The direct comparison of the dispersion energies computed for the first two singlet states by the LRD and QM/MMPol methodologies is reported in Figure 3. This comparison clearly shows the good agreement between the LRD and the cLR^3^ schemes: the general trend of increasing the dispersion energy in passing from GS to the *n*-π* and π–π* states is reproduced, with differences less than 10 cm^–1^ (less than 1%) for the ES dispersion energy values of the Pyridine-MeNH_2_ dimer and less than 280 cm^–1^ in the case of the uracil-cyclopentane one (around 10%). These values are particularly good if one also takes into account that a different functional was used for the two methods. Instead, the simple use of the unscaled TS scheme applied to excited states systematically underestimates the dispersion energies of both transitions in the two systems.

**Figure 3:**
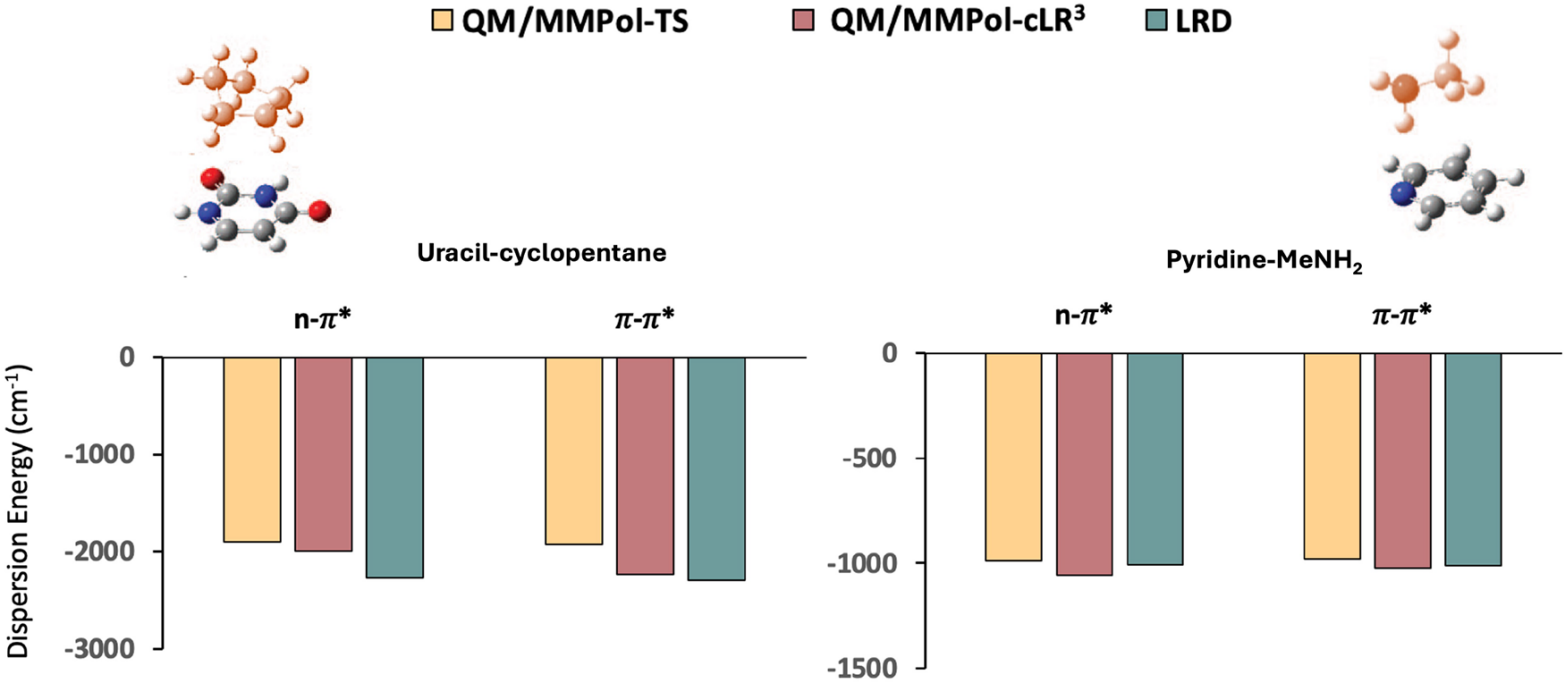
ES dispersion energies (cm^–1^) of S_1_ (*n* → π*) and S_2_ (π → π*) transitions of uracil in the presence of cyclopentane (left) and pyridine in the presence of MeNH_2_ (right). MM chromophores are highlighted in red. QM/MMPol-TS refers to the SS-dispersion term obtained without the *ad hoc* excited-state scaling procedure.

### Azulene Solvatochromism: A Case Dominated by the Excited-State Polarizability

We now consider the solvatochromism of azulene. The first two singlet transitions of azulene (usually labeled as L_b_ and L_a_ according to Platt’s nomenclature^68^) are known to show an opposite solvatochromism in low-polarity solvents like CCl_4_.^7^ The upper part of Figure 4 shows the opposite solvatochromisms of L_b_ and L_a_ states: the QM/MMPol-cLR^3^ model not only reproduces the experimental trend qualitatively but also quantitatively.^7^ Differences are around 28 and 8 cm^–1^ for L_b_ and L_a_, respectively.

**Figure 4:**
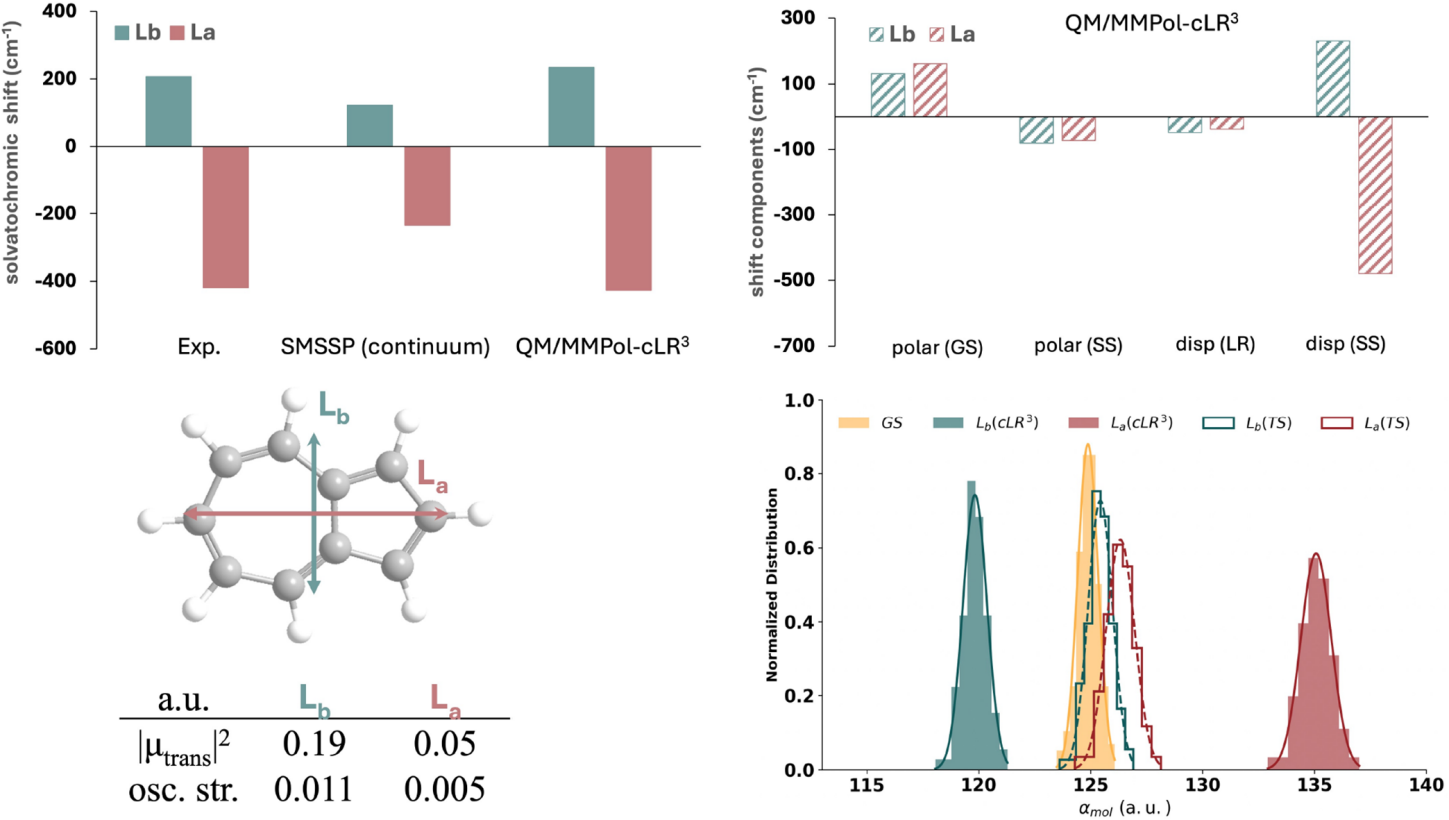
MD-averaged QM/MMPol-cLR^3^ excited-state properties of azulene in CCl_4_. Top left: gas-to-solvent solvatochromic shift (cm^–1^) of the first two singlet excited states of azulene in CCl_4_. Experimental values are taken from^7^. SMSSP refers to the empirical continuum approach of Marenich et al.^29^ Top right: the four energetic components of the QM/MMPol-cLR^3^ approach are reported: polarization frozen to GS, state-specific polarization, linear response dispersion-like term, and state-specific dispersion. Bottom left: QM/MMPol-cLR^3^ dipolar and oscillator strengths (a.u). Bottom right: normalized distribution of molecular polarizabilities obtained as the sum of atomic contributions, using the scaled (QM/MMPol-cLR^3^) and unscaled (QM/MMPol-TS) approaches.

In Figure 4, we dissect the different contributions to the solvatochromic shift of azulene. The leading term is SS dispersion, which is linked to the changes in molecular polarizability upon excitation. Instead, the total polarization and LR terms are small and tend to cancel each other. We recall that the LR term is a part of the dispersion energy due to the environment’s fluctuations^35^ and depends on the transition density: the weak transition dipoles of L_a_ and L_b_ transitions of azulene thus explain the small LR-disp values observed. Our results are in agreement with the usual assumption^5^ in the literature that the solvatochromic shifts due to bulk electrostatic effects for benzene, azulene, naphthalene, anthracene, and coronene are small.^7,29,69^ The solvatochromism due to dispersion normally induces a red shift in many organic chromophores, since very often excited-state polarizabilities are larger than ground-state ones (i.e., α_mol_^ES^ > α_mol_^GS^). In the L_b_ state of azulene, however, we can observe the opposite situation (Table 1). This highlights the crucial role of the atom-dependent rescaling included in the QM/MMPol-cLR^3^ procedure to recover the full QM results: as the polarizabilities of the different electronic states cannot be reproduced from the effective atomic volumes,^39^ a procedure to recover the correct ones is essential to calculate the SS-dispersion contribution to electronic transitions.

### BChl-a Solvatochromism: A Fine Interplay of Excited-State Polarizability and Transition Density

There has been an ongoing debate regarding the role of dispersion effects in solvatochromism and whether the associated shift is influenced by the oscillator strength of the transition, which has generally been used to explain experimental data.^5,69^ In 2008, Renger and coauthors^28^ introduced a state-specific semiempirical expression for the shift in transition energies of molecules in nonpolar solvents in terms of the difference in mean-square transition dipole moments, mapped onto the solvation energy of an extended unit dipole in a spherical cavity embedded in a continuum dielectric medium. This expression was used to explain the solvatochromism of the two lowest electronic excitations (Q*_y_* and Q*_x_* transitions) of bacteriochlorophyll a and bacteriopheophytin a in different nonpolar solvents. This model implies that there is always a red shift for nonpolar solutes in nonpolar solvents, which is contradictory, for example, with the experimental blue shift discussed in the previous section for the L_b_ state of azulene. To solve the conundrum of the origin of the solvatochromic shift of a nonpolar system in a nonpolar environment, we applied the QM/MMPol-cLR^3^ model to BChl-a in a CCl_4_ solution. Indeed, the BChl-a system, in contrast to azulene, shows significant transition dipole strengths, and we can show here that both the ground to excited-state polarizability change and the transition density contributions play a significant role that can be opposite. Solvatochromic contributions for this system are dissected in Figure 5, where we show that different terms contribute to the observed solvatochromic shifts of both Q_*y*_ and Q_*x*_. First, even if the SS-polarization term is nearly negligible (around 10 cm^–1^), the polarization due to the GS is large and comparable to that of the dispersion terms. Indeed, the energies computed with GS frozen polarization or including SS polarization are very similar. In contrast, dispersion affects the energies differently for the Q_*y*_ and Q_*x*_ states, resulting in positive and negative corrections, respectively. As is the case with azulene, the changes in molecular polarizability upon excitation explain the sign of the SS-dispersion term: a smaller ES polarizability compared to that of the GS leads to a blue shift. On the other hand, the weaker transition dipole of Q_*x*_ compared to the Q_*y*_ state explains the smaller LR dispersion shift obtained. The QM/MMPol-cLR^3^ results quantitatively reproduce the experimentally estimated solvatochromic shift of both transitions with only a slight underestimation of Q_*y*_, capturing the correct shift direction. The inclusion of the SS dispersion is crucial here, and experimental trends are recovered only when both SS and LR dispersion terms are accounted for in the model.

**Figure 5:**
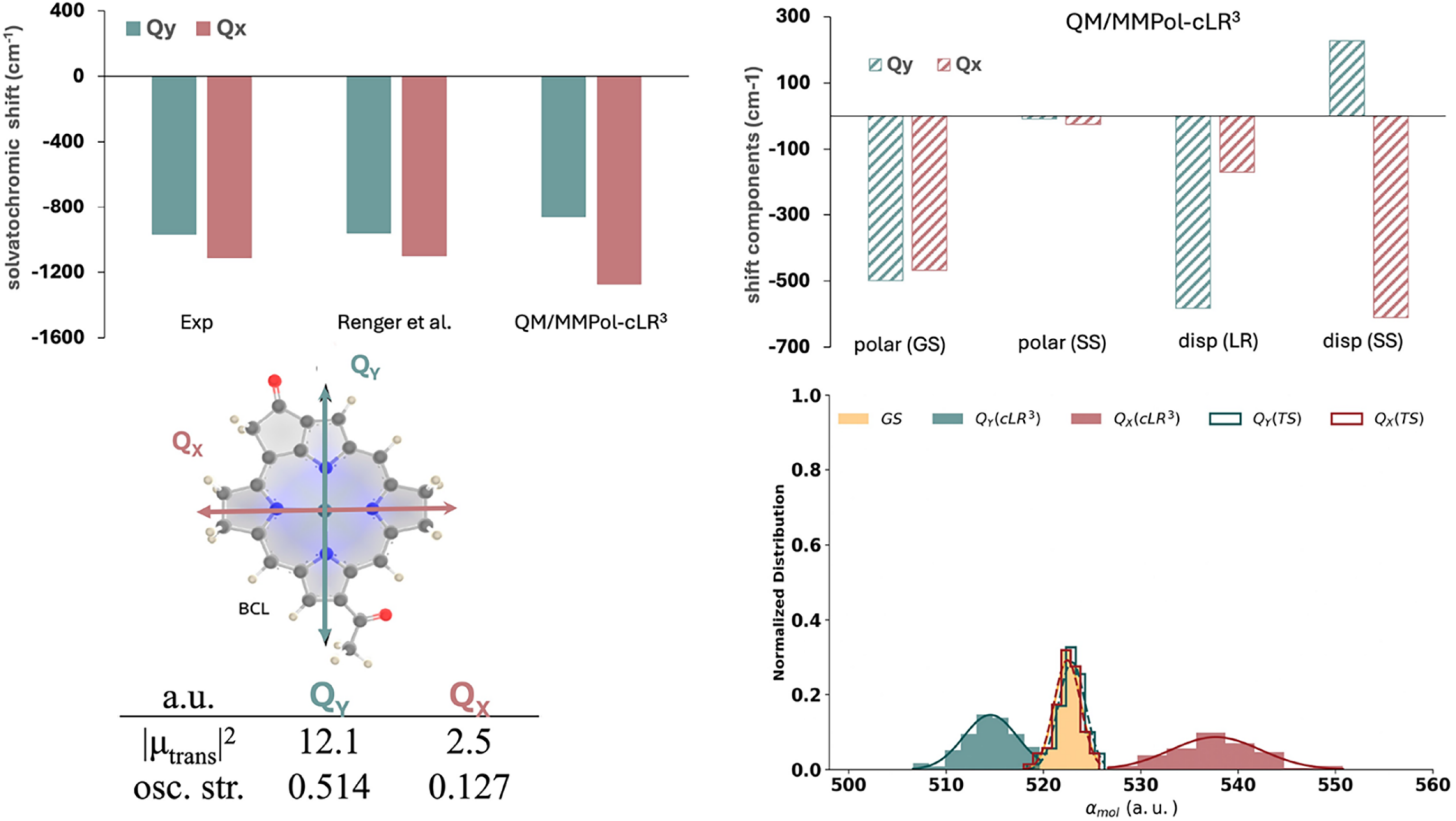
MD-averaged QM/MMPol-cLR^3^ excited-state properties of BChl-a in CCl_4_. Top left: gas-to-solvent solvatochromic shift (cm^–1^) of the first two singlet excited states of BChl in CCl_4_. Experimental values are obtained by extrapolating the gas-phase BChl-a transitions;^70^ Renger et al. refers to the continuum approach parametrized by fitting the experimental excitations.^28^ Top right: the four energetic components of the QM/MMPol-cLR^3^ approach are reported: polarization frozen to GS, state-specific polarization, linear response dispersion-like term, and state-specific dispersion. Bottom left: the QM/MMPol-cLR^3^ dipolar strength and oscillator strength are reported (a.u). Bottom right: normalized distribution of molecular polarizabilities, obtained as the sum of atomic contributions, using the scaled (QM/MMPol-cLR^3^) and unscaled (QM/MMPol-TS) approaches.

### BChl-a in the LH_2_ Complex: Dispersion Effects Depend on Local Environment

The LH2 complex is a fundamental constituent of the photosynthetic unit of purple bacteria and displays a symmetric ring structure based on a common repetitive α–β subunit formed by two transmembrane α helices, the α- and β-aproprotein, 3 BChls, and a carotenoid. In *R. acidophilus*, The LH2 complex exhibits a 9-fold rotational symmetry and contains a total of 27 BChl molecules arranged in two rings of different sizes (Figure 2, bottom). The smaller ring, composed of nine BChls, gives rise to the absorption band centered at 800 nm (the B800 ring), while the larger ring, consisting of 18 BChls, is responsible for the absorption at 850 nm (the B850 ring). In the B800 ring, the BChl molecules are relatively far apart and thus only weakly coupled, whereas in the B850 ring, the closer packing of the 18 BChls leads to strong excitonic coupling. This stronger coupling, together with pigment–protein interactions, accounts for the red shift of the B850 absorption band relative to that of B800.^40,41^ In previous studies, we applied an excitonic polarizable QM/MM approach to LH2 and showed that the differences in exciton structure at low and high temperatures are mainly related to fluctuations in the couplings between the Q*_y_* states of the BChls,^41^ whereas the mixing between excitons and charge transfer states in the B850 ring is responsible for the modulation of position and broadening of absorption bands.^42^ Here, we use the QM/MMPol-cLR^3^ model to investigate the role of dispersion in tuning the Q_*y*_ excitation (site) energies of BChls in LH2. Due to the symmetry of the system, the 27 BChls can be classified into three types, which differ in terms of local environment: B850α, B850β, and B800γ, where the first two belong to the B850 ring and the third to the B800 ring. The results shown in Table 2 follow the same physical trend as those obtained for BChl-a in CCl_4_ solution: the site energies of BChls in LH2 are modulated by ground-state polarization and dispersion interactions. The values for the B850α, B850β and B800γ pigments span on average a range of 242 cm^–1^, which impacts the energy funnel characterizing the LH2 function. 70% of this range is due to the ground-state polarization of the environment (Δ*E*_GS–pol_), whereas the remaining 30% originates from pigment-dependent dispersion effects, specifically from the interplay between SS and LR dispersion contributions that differ according to the local pigment environments. Importantly, omitting the SS-dispersion term would lead to an overestimation of the total dispersion effect since SS and LR components have opposite signs and partially cancel each other. In Figure 6, we illustrate the origin of these differences between B850α, B850β, and B800γ. We color the residues surrounding each pigment according to their contribution to the *R*_LR–disp_ and *R*_SS–disp_ terms. Note that these values correspond to a particular structure extracted from the MD trajectory, so they differ from the MD averages reported in Table 2. Overall, the B850 pigments present larger dispersion shifts (both SS and LR terms) than BChl B800γ, which can be explained by the short interpigment separations among the quasistacked BChls in the B850 ring. Indeed, B850α displays the largest contributions with neighboring B850β BChls and with its coordinating His. B850β also interacts with its B850α neighbors, as well as with B800γ. In contrast, B800γ BChls display smaller dispersion interactions mostly with B850β. Finally, it is interesting to note that MD-averaged SS dispersion terms, reported in Table 2, amount to relevant values as large as 230 cm^–1^ for B850 and 200 cm^–1^ for B800, which are on the same order of magnitude as the corresponding exciton couplings between bright states,^41^ or even larger than couplings between local and CT excitations.^42,60^ These findings indicate that dispersion interactions play a significant role in modulating the environmental influence on excitonic parameters, which are key to rationalizing the light-harvesting properties of antenna complexes.

**Figure 6:**
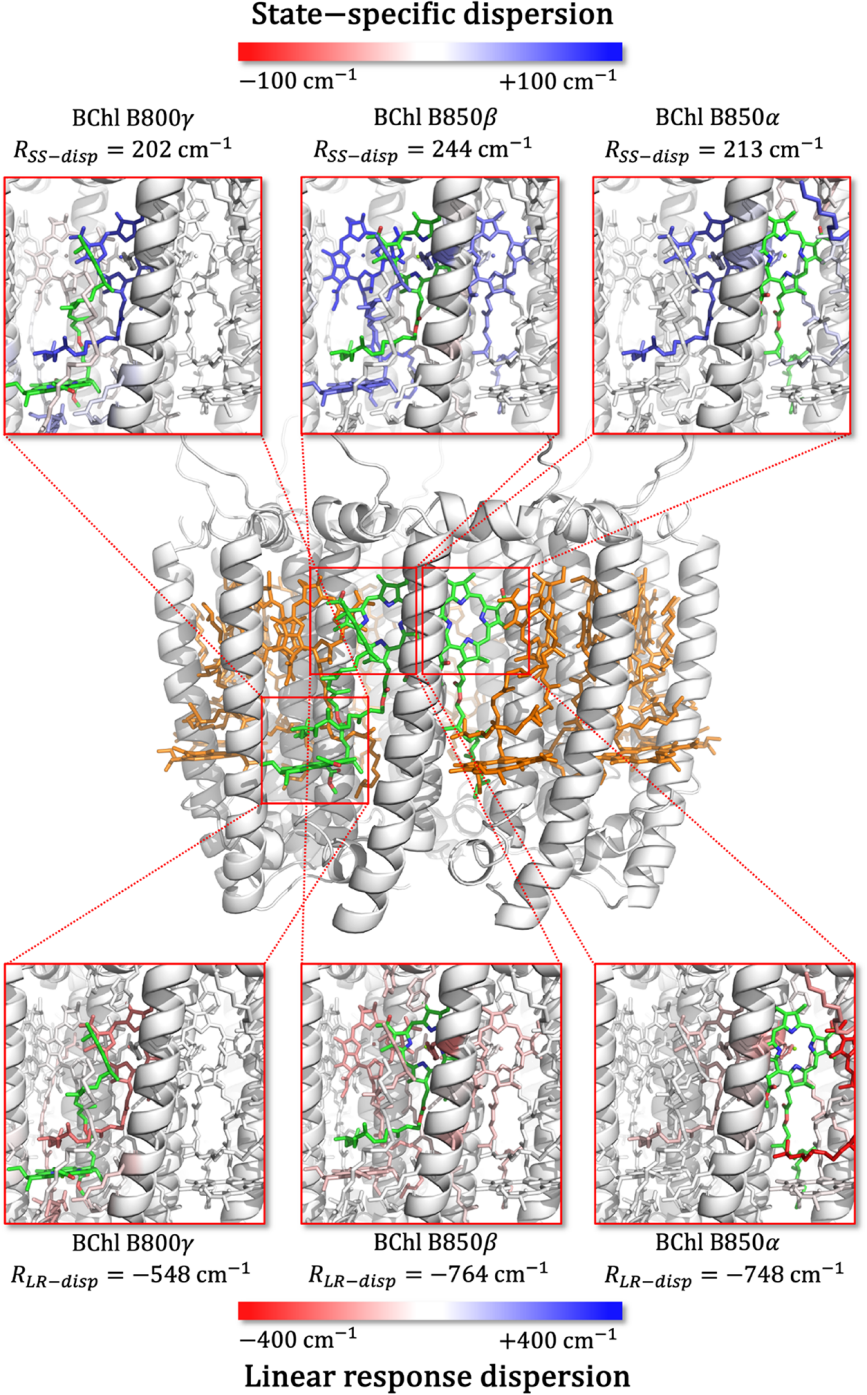
Illustration of residue contributions to the *R*_LR–disp_ and *R*_SS–disp_ dispersion shifts for the B850α, B850β, and B800γ BChl repeating units in the LH2 complex. Residues are colored according to their contribution to the total QM/MMPol-cLR^3^ dispersion shift computed for a structure extracted from the MD trajectory.

**Table 2:** Dissection of MD-Averaged Energetic Terms to the Q_*y*_ Site Energies of the B850α, B850β, and B800*γ* BChl-a Pigments in the LH2 Complex^a^

	BChl type	Δ*E*_QM/MMPol–cLR^3^_	Δ*E*_GS–pol_	*R*_SS–pol_	*R*_LR–disp_	*R*_SS–disp_
B850 ring	α	13722	14214	–14	–714	235
	β	13816	14295	–14	–699	235
B800 ring	γ	13964	14385	–13	–611	204

a Data are given in cm^–1^.

## Conclusions

We have presented QM/MMPol-cLR^3^, an embedding model that explicitly accounts for dispersion and polarization effects in electronically excited states by combining state-specific polarization, linear-response dispersion, and state-specific dispersion contributions. This framework disentangles the different physical origins of excited-state solvatochromism, enabling us to rationalize the spectral shifts. We have here applied the model to explain the opposite spectral shifts observed for the two lowest transitions in azulene and the selective modulation of Q_*x*_ and Q_*y*_ bands in bacteriochlorophyll a. We also showed that the model captures pigment-dependent dispersion shifts in the LH2 light-harvesting complex, directly influencing site-energy differences that control excitation-energy transfer.

Taken together, QM/MMPol-cLR^3^ provides a physically grounded and computationally efficient route to include excited-state dispersion in large molecular environments with atomistic detail. This allows us to investigate the multiple physical mechanisms underlying solvatochromic shifts well beyond the case of isotropic solvents and to resolve a longstanding conundrum in their interpretation. As such, it establishes a general and transferable strategy for studying light-induced processes in complex systems, from solvated chromophores to biomolecular aggregates and molecular materials.

## Supplementary Material



## Notes

Effect of the environment used to compute the HHS atomic polarizabilities, the exchange–correlation functional, and the basis set on the QM/MMPol-cLR^3^ solvatochromic shifts of azulene in CCl_4_ is reported for a randomly selected frame from the classical MD simulation (PDF)
