# Supplementary material for: How Dispersion Interactions at the Excited State Can Tune Photochromism of Embedded Chromophores
Source: J Am Chem Soc. 2025 Dec 24;148(1):1847–57. doi: 10.1021/jacs.5c19241 (PMC12814335; doi:10.1021/jacs.5c19241)
Supplement: Supplementary file 1 [file ja5c19241_si_001.pdf]

Supporting Information for How Dispersion Interactions at the Excited State Can Tune Photochromism of Embedded Chromophores

Ciro A. Guido[1,*], Lorenzo Cupellini[2], Benedetta Mennucci[2], Carles Curutchet[3,4,†]

E-mail:

[1]Dipartimento di Scienze e Innovazione Tecnologica, Università del Piemonte Orientale, Viale T. Michel 11, Alessandria 15121, Italy.

[2]Dipartimento di Chimica e Chimica Industriale, Università di Pisa, Via G. Moruzzi 13, Pisa 56124, Italy.

[3]Departament de Farmàcia i Tecnologia Farmacèutica, i Fisicoquímica, Facultat de Farmàcia i Ciències de l'Alimentació, Universitat de Barcelona (UB), Barcelona 08028, Spain.

[4] Institut de Química Teòrica i Computacional (IQTCUB), Universitat de Barcelona (UB), Barcelona 08028, Spain.

*ciro.guido@uniupo.it; †carles.curutchet@ub.edu

Abstract

The Supporting Information reports an analysis of how HHS atomic polarizabilities, the exchange–correlation functional, and the basis set affect the QM/MMPol-cLR^3 solvatochromic shifts of azulene in CCl_4. Additional plots with numerical comparisons and computational details are provided to clarify the robustness of the method.

Contents

List of Figures

S1 Solvatochromic shift of azulene in CCl_4 . . . . S4

Effect of HHS atomic polarizabilities, xc-functionals, and basis set

To address the sensitivity of the QM/MMPol-cLR^3 approach to the underlying computational ingredients, we evaluated the impact of (i) the environment used to compute the HHS atomic polarizabilities, (ii) the exchange–correlation functional, and (iii) the basis set. All comparisons were performed on the same randomly selected MD frame of azulene in CCl_4, and the resulting shifts are reported in Fig. S1.

First, we examined whether the HHS atomic polarizabilities should be determined in the MMPol environment or in the gas phase. Moving from gas-phase HHS polarizabilities to those computed in an MMPol representation of CCl_4 leads to limited variations of the cLR^3 solvatochromic shift, namely 44 cm^{-1} for the L_b state and 55 cm^{-1} for L_a. These results indicate that the environmental dependence of the HHS polarizabilities has only a minor influence on the predicted solvatochromism for this system: this is expected, since azulene is a quite rigid chromophore and its geometry has been kept frozen during the classical MD simulation.

Second, we assessed the sensitivity to the exchange–correlation functional. The variation is again modest when moving from M062X to CAM-B3LYP (20 cm^{-1} for L_b and 50 cm^{-1} for L_a), consistent with the similar fraction of exact exchange in the two functionals (54 % for M062X and from 19 to 65 % for CAM-B3LYP). Reducing the exact-exchange content with PBE0 leads instead to a more marked decrease of the solvatochromic shift, on the order of 100 cm^{-1} for both excited states, in line with previous observations of fine interplay between state-specific environment response approaches and the xc-functionals used.[1]

Finally, changing the basis set from a split-valence double-ζ (6-31+G*) to a triple-ζ (6-311+G*) induces only minor effects: 40 cm^{-1} for L_b and as little as 8 cm^{-1} for L_a.

Overall, although quantitative variations are observed when individual ingredients are modified, the differential solvatochromism between the two excited states is robustly preserved.

These tests also clarify the best practice: using HHS atomic polarizabilities computed in the gas phase is sufficient for a rigid system in a isotropic environment, but in general we suggest to derive the β_A scaling factors in the given specific environment especially if anisotropic, or if the chromophore is quite flexible, as we did for the BChl-a in the main text.

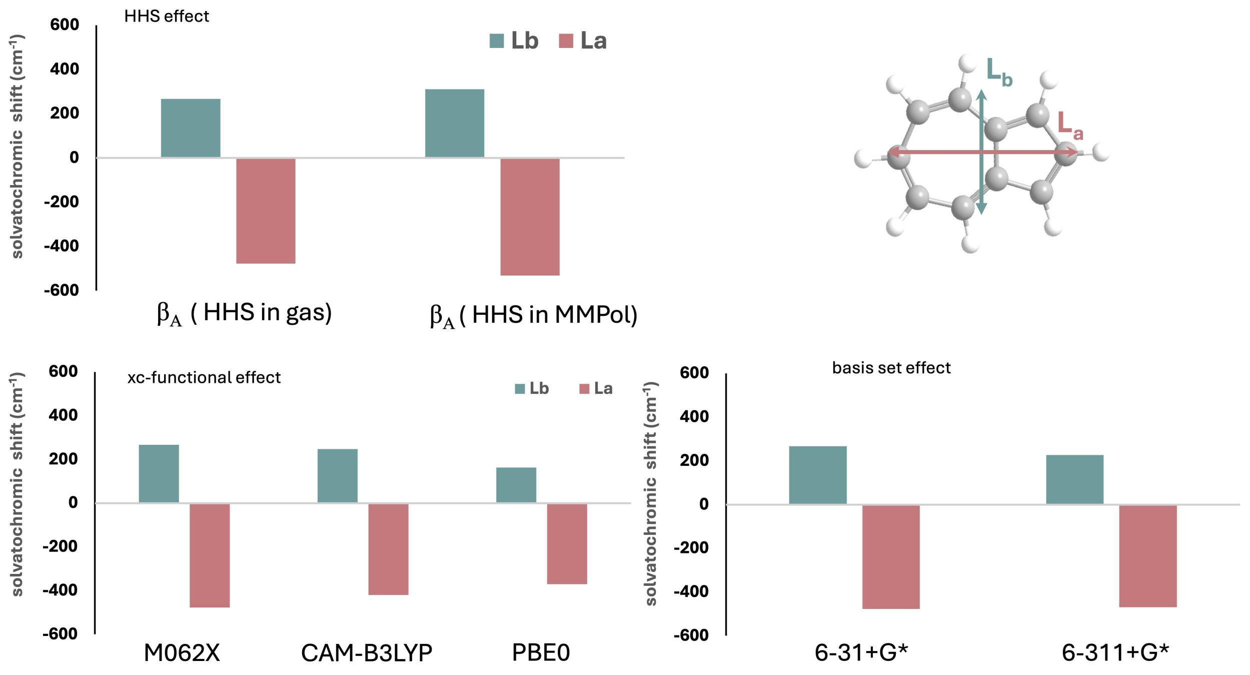

Fig. S1: Solvatochromic shift of azulene in CCl_4 for a randomly selected frame. Top, left: effect of determining the scaling factors β_A from HHS atomic polarizabilities computed for the chromophore in gas phase or in a MMPol solution of CCl_4; bottom, left: effect of xc-functional used to determine energies and β_A scaling factors (HHS gas phase); bottom, right: effect of basis set used to determine energies and β_A scaling factors (HHS gas phase).

References

(1) Guido, C. A.; Jacquemin, D.; Adamo, C.; Mennucci, B. Electronic Excitations in Solution: The Interplay between State Specific Approaches and a TD-DFT Description. *J. Chem. Theory Comput.* **2015**, *11*, 5782–5790.
